# Mindfulness influence on psychological wellbeing: in search of cultural adaptations

**DOI:** 10.3389/fpsyg.2025.1550949

**Published:** 2025-05-27

**Authors:** Reut Paz, Nitza Davidovitch

**Affiliations:** Department of Education, Ariel University, Ariel, Israel

**Keywords:** mindfulness, teachers, psychological wellbeing, cultural adaptations, quality of life, cultural sensitivity, identity and resilience

## Abstract

**Introduction:**

In the last decade, mindfulness-based programs have been assimilated among educational staff to cultivate socio-emotional skills and mental resilience. Most mindfulness programs use generic international models, omitting explicit acknowledgement of cultural and ethnic differences. Recent research highlights the importance of considering participants’ identities and diverse cultural and religious needs. The first aim of the current study is to explore whether mindfulness affects psychological well-being among Orthodox Jewish teachers. Additionally, the study examined the relationship between mindfulness as a personality trait and psychological well-being and resilience among Orthodox Jewish teachers. The second aim is to examine whether there is a tension in practicing mindfulness among participants who are Orthodox educators, and whether there is a need for cultural adaptations for religious populations to increase the effectiveness of the program.

**Methods:**

The participants in this pioneer study were Orthodox teachers and principals from state religious schools who participated in the mindfulness program. The questionnaires were distributed among Orthodox teachers to characterize the group on several measures related to psychological well-being and resilience, as well as on measures of religiosity, to clarify whether there is an association between deep-rooted religious foundations and perceived mental resilience.

**Results:**

The research findings indicate that trait mindfulness and mindfulness training are related to increased psychological well-being and resilience. Trait mindfulness was significantly negatively associated with perceived stress. The study also showed that ultra-Orthodox teachers reported a higher sense of stress than did national religious teachers.

**Discussion:**

It is evident from the current study that mindfulness qualities may support resilience and psychological well-being among Orthodox educators, whereupon it is important to nurture these qualities among them via mindfulness-based professional development. Moreover, it is evident from the study that to increase the efficacy of these programs, it is necessary to be aware of the participants’ ethnic, religious, and cultural features.

## Introduction

The concept of mindfulness is known to denote mindful awareness and attentiveness. Dahl et al. (2015) defined mindfulness as a psychological skill that includes processes of self-regulation and attention, aimed at the present-moment experience and characterized by inquisitiveness, openness, and acceptance (Dahl et al., 2015). Kabat-Zinn (1994) defined mindfulness as a conscious state that arises from paying attention to studying one’s physical and mental experience of the present non-judgmentally.

Mindfulness practice originates from the Buddhist tradition. The method is rooted in meditative practices utilized for millennia. In the last 40 years, these ideas arrived in the West, where they were absorbed and grounded as intervention methods aimed at clarifying and exploring the present moment (Kabat-Zinn, 1994). One well-known method is Mindfulness-Based Stress Reduction (MBSR), developed in the 1970s by Kabat-Zinn (2003), which served as the basis for other therapeutic methods, significantly documented and established in the research literature (for a review of the different methods see Levit-Binnun and Arbel, 2021). In recent years, there has been growing interest in mindfulness, both in the clinical field (medicine and psychology) in academia and the educational field, and even in hi-tech and business management (Desai, 2024; Godfrin and van Heeringen, 2010).

### Distinction and interrelation between trait mindfulness and mindfulness practice

There is a distinction between mindfulness as an inherent personality trait—one’s natural capacity to relate to the present everyday moment without being immersed in thoughts and annoyance (Brown et al., 2007) and the application of mindfulness through practice. Mindfulness defined as a conscious state that arises from paying attention to studying one’s physical and mental experience of the present non-judgmentally (Kabat-Zinn, 1994). We learn to identify the present experience: the bodily and sensory feelings, the emotional tone, and the cognitive processes. Mindfulness is described as respectful and non-judgmental awareness. Previous studies have demonstrated that mindfulness practice enhances dispositional mindfulness, indicating a close and dynamic relationship between the two (Baer et al., 2008; Carmody and Baer, 2008; Zhang et al., 2023).

### The association between trait mindfulness and psychological wellbeing in the research field

In recent years, studies have shown that differences in psychological wellbeing are associated, among other things, with differences in people’s ability to be in a state of mindful awareness of the here and now. Studies have consistently proven a link between high levels of mindfulness as a trait and psychological wellbeing. For instance, trait mindfulness has been linked to quality of life (Godfrin and van Heeringen, 2010) and to general psychological wellbeing (Shahar et al., 2010; Shapiro et al., 2008). In addition, people who described good mindfulness competence also reported higher psychological wellbeing and a decline in strain, anxiety, and depression (Hofmann et al., 2010; Carmody and Baer, 2008).

Mindfulness practice has been found to reduce stress and negative feelings, improve one’s state of mind and sense of psychological wellbeing, improve the ability to manage attention and encourage prosocial behavior (Goleman and Davidson, 2017). Mindfulness approaches and methods aimed at nurturing accepting awareness of the present moment (Bishop et al., 2004; Brown and Ryan, 2003; Kabat-Zinn, 1990) can improve the regulation of emotions and the symptoms underlying anxiety and depression, as well as increasing one’s positive state of mind (Goyal et al., 2014; Hofmann et al., 2010).

Mindfulness programs are presented as practical tools that enhance emotional regulation, personal resilience, and adaptability in environments characterized by volatility, uncertainty, complexity, and ambiguity (VUCA) (Essens et al., 2023).

### Mindfulness and cultural sensitivity

Most of the research on mindfulness has been conducted with the population of Europe and the United States, with a western socioeconomic and cultural characterization, and does not include diverse populations (Hathaway and Tan, 2009). Recently, there is a growing understanding that mindfulness should be adapted more specifically to different groups. In the last decade, mindfulness-based interventions adapted to the individual’s religious setting have been developed to promote this intervention among religious populations as well (Trammel, 2018).

A study conducted among ultra-orthodox women in Israel examined the association between MBSR and religiosity. Cultural adaptations of the MBSR program for ultra-orthodox women were proposed. Most of the participants (93.33%) found that attuning the contents to the Jewish outlook and adding religious sources enriched the program and increased the ability to connect with the world of mindfulness (Kolsky, 2022).

### Mindfulness, education, and religious identity

The purpose of mindfulness in education, as viewed by teachers, is to support the multiple common challenges of teaching such as stress, strain, and burnout. Stress can be manifested in sleeplessness, lack of empathy toward students and other staff members at the school, lack of self-efficacy, difficulty managing tasks on time, and others. Over time, these may lead to burnout and early retirement from the profession (Jennings and Greenberg, 2009). Teachers in the state religious school system are from the national religious and ultra-orthodox sectors. Ultra-orthodox society is separate and comprised of closed communities, with the aim of preserving its characteristics and reducing the influence of modern society as much as possible. This society is characterized by values of modesty and strict observance of all religious precepts. It is committed to halakhic traditions and frameworks in general and Torah study for men in particular (Friedman, 1995).

In contrast, national religious society was established in Eastern Europe in the 19^th^ century under the name Hapoel Hamizrahi. Its guiding principle is the connection between religious values and national values and its motto is a balance between Torah and work. It willingly accepted involvement in civic duties, such as the enlistment of its sons and daughters in military service and national service. The national religious movement began as a revolutionary movement, manifested in the establishing modern values as part of its spiritual foundation. National religious society lives in a tension between involvement in the modern national reality and maintaining separate religious integrity. The dialectical tension between tradition and modernity, underlying the ideological foundation of the national religious movement, was resolved by Torah-oriented groups within it by giving unequivocal priority to tradition (Arnon et al., 2012).

Previous studies have shown more psychological wellbeing among people with religious faith (Green and Elliott, 2010; Witter et al., 1985), as well as less depression and anxiety (Braam and Koenig, 2019), even during the time of COVID-19 (Kimhi et al., 2021). In a study among ultra-orthodox women, which examined the association between MBSR and faith, some of the research aims were to examine whether and what level of religiosity moderates the association between mindfulness and self-compassion personality traits and measures of mental health. The findings showed a significant positive association between well-developed mindfulness competencies and psychological wellbeing, as well as a negative association with strain, depression, and anxiety (Kolsky, 2022).

Considering the lack of comprehensive research examining the impact of mindfulness within the Orthodox Jewish educational sphere, and due to the unique complexity arising from the interaction between mindfulness practices and religious identity, this study constitutes an innovative and significant contribution—both theoretically and practically—to the development and implementation of culturally adapted programs for religious populations.

## Materials and methods

### Research aims

From the literature review, it is evident that while there are studies on mindfulness and education (Black et al., 2012; Jennings and Greenberg, 2009), there is a dearth of studies that focus on mindfulness in religious-oriented schools. The current study examines how mindfulness is perceived by Orthodox Jewish teachers and whether they experienced difficulty or sensitivity when exposed to mindfulness content or during personal or group practice. In addition, we examined whether they formed intuitive connections between the world mindfulness and the Jewish world and whether there was of a need for cultural adaptations to help them implement the practice as an integral part of their life routine, to nurture their wellbeing and mental resilience.

This study has two aims and was conducted by mixed methods: quantitative and qualitative. The first research aim was to explore whether there is a connection between mindfulness and mental resilience and wellbeing, similar to the findings of other studies in this field. The second aim was to explore whether there is tension involved in practising mindfulness, which originates from the Buddhist tradition, among participants who are Orthodox educators, and whether there is a need for cultural adaptation for religious populations to increase the program’s effectiveness.

### Research design of the quantitative study

The study was conducted before and after the implementation of a mindfulness program operated by the Sagol School. The Sagol program was developed and based on the “whole school approach,” aimed at developing in schools a space for developing and flourishing using tools and approaches of socioemotional learning and contemplative pedagogy, based on mindfulness and neuroscience. The program is implemented at schools throughout Israel by instructors from the Sagol School, who come to the schools and hold a professional development plan for teachers participating in the program.^1^ The first year of the “Introduction to Mindfulness and Neuroscience” includes acquaintance with terminology and language from the domain of socioemotional learning (also called “the Sagol culture”), training principals and a team of leading teachers via a course on mindful leadership, individual and group accompaniment of the principals and the team of leading teachers, and initial assimilation of the contents in the school routine. The second year of the plan includes advanced training aimed at developing and assimilating Sagol’s pedagogic practices in the classrooms and regular school conduct patterns in the spirit of the “the Sagol culture,” developing a mindful community around the school.

### Research procedure

The quantitative study examined the association between mindfulness as a personality trait, perceived stress, and various indicators of mental wellbeing and psychological resilience.

The questionnaires were distributed among Orthodox Jewish teachers to assess their traits across several indices measuring mindfulness, psychological wellbeing, psychological resilience and others measuring religiosity (Psychological resilience was assessed through the perceived stress and mindfulness questionnaires, which included measures of psychological wellbeing and coping ability with stress).

### Research questions

- Is mindfulness as a personality trait associated with psychological well-being and resilience among Orthodox Jewish teachers?
- Is mindfulness as a personality trait associated with religiosity?

### Research sample

The research participants consisted of 87 teachers and principals teaching in the state religious school system, in the age range of 20–55, affiliated with national religious society or ultra-orthodox society, who had begun and finished the first year of a mindfulness program part of a three-year mindfulness program assimilated in schools by the Sagol laboratory. Before completing the questionnaires, the participants signed an online consent form.

### Research tools

- Demographic questionnaire: the questionnaire consisted of 13 questions on marital, religious, and employment status.
- Religiosity questionnaire (Hamada, 2002): the questionnaire was intended to examine the level of religiosity and of religious faith. It was comprised of 3 closed-ended questions on one’s level of devotion to one’s religion and beliefs.
- Mindfulness questionnaire: mindfulness was measured by the commonly used Freiburg mindfulness inventory (FMI) (Walach et al., 2006), which includes 14 items. For example: “I am open to the experience of the present moment”; “When I notice an absence of mind, I gently return to the experience of the here and now.” In the current study, Cronbach’s alpha reliability coefficient was 0.84.
- Perceived stress scale (PSS) (Cohen and Williamson, 1988). The scale is comprised of 14 items measuring stress. Each item is ranked from 1 “never” to 4 “often.” The score on the questionnaire is comprised of the sum of all items. In the current study, Cronbach’s alpha reliability coefficient was 0.90.

### Analysis of the research data

Analysis of the data from the questionnaires and the statistical processing were carried out with SPSS program version 25. After coding the variables and receiving optimal internal reliability, the theoretical variables mentioned in the research hypotheses were calculated. At the stage of initial analysis, descriptive statistics were explored regarding the participants’ personal and demographic background variables. In the deductive part of the findings, the correlations between the main research variables and the personal background characteristics were first examined using a Pearson’s test. To examine the two quantitative research questions, an independent samples *t*-test was utilized. The mediation model in the third hypothesis, which explored differences in the main research variables by religiosity, was analyzed using the PROCESS tool (Model 4).

### Research design of the qualitative study

#### Research questions

- Did any sensitivity emerge or is there an inner dissonance concerning faith and culture among the religious teachers who participated in the mindfulness program?
- Did they find connections between the content world of mindfulness and the Jewish religious content world?
- In their view, is it necessary to adapt the program for their identity as Orthodox women?
- What is the effect of the practice on the teachers’ psychological wellbeing?

#### Research population

The participants in the qualitative study had also participated in the quantitative component of the research. The current study included 3 principals and 11 teachers. Sixteen interviews were held (two teachers were interviewed twice, at the end of the first year and at the end of the second). The participants were homeroom and subject teachers in the first to sixth grades and principals, aged 20–55. Most of the participants were ultra-orthodox, from central Israel, who were taking part in a professional development plan within the program operated by the Sagol program.

### Research tools

Semi-structured interviews were used to examine whether the practice is beneficial, whether there was cultural sensitivity, and whether there is room for unique adaptation of mindfulness in the educational field. Part of the questions were: “Were there any specific aspects of the program that proved beneficial for you as an educator?,” “Can you describe a moment/case when the program’s content aligned well with your personal belief or religious idea/values?” “How did the program fit into your world on a personal level? Were there things that contradicted your beliefs or values? In the next stage, thematic analysis of the interviews was conducted, with the aim of identifying the main themes.

### Research procedure

The analysis process was thematic. Part of the analysis followed the six steps proposed by Braun and Clarke (2012): familiarization with the data, generating codes, combining codes into themes, reviewing themes, determining the significance of themes and naming them, and creating a set of themes that refer to the research questions. The second part on the impact of mindfulness practice on the Orthodox teachers was carried out through grounded thematic analysis to identify, analyze, and report patterns emerging from the data.

## Results

### Research findings of the quantitative study: association between trait mindfulness and psychological wellbeing and psychological resilience

Table 1 presents the demographic characteristics of the research participants. Almost half the teachers were ultra-orthodox and the rest were national religious.

**Table 1:** Demographic characteristics of all teachers in the research sample (*N* = 87).

Variable	Values	*N*	%	M	SD	Range
Religiosity	National religious	46	52.9			
	Ultra-Orthodox	41	47.1			
Role at the school	Homeroom teacher	68	78.2			
	Subject teacher/auxiliary teacher	21	24.1			
	Coordinator	25	28.7			
	Special education teacher	12	13.8			
	Teaching assistant	2	2.3			
	Management staff	8	9.2			
	Other	4	4.6			
Extent of position at the school	61–80%	9	10.3			
	81–100%	78	89.7			
					
	Considerably below average	35	41.2			
	Below average	22	25.9			
	Average	18	21.2			
	Above average	9	10.6			
	Considerably above average	1	1.2			
Age of the teachers				18.43	9.99	5–40
Number of individuals in the household				5.38	2.08	1–11
No. of weekly teaching hours				20.95	5.40	4–36

Role at the school reaches a total of more than 100% because multiple answers could be given to the question.

Table 2 presents the correlations between the main research variables and the personal background characteristics. Examination of these associations produced one significant negative correlation between length of professional experience and teachers’ perceived stress (*r* = −0.23, *p* < 0.05). The greater the teachers’ length of experience in teaching, the lower their perceived stress. The findings also showed that trait mindfulness was significantly negatively correlated with perceived stress (*r* = −0.56, *p* < 0.001), such that teachers with high levels of trait mindfulness reported lower levels of stress.

**Table 2:** Correlations between the main research variables and professional and demographic background characteristics (*N* = 87).

	1	2	3	4	5	6	7	8	9
Trait mindfulness	–	−0.559^***^	−0.003	0.159	−0.155	0.049	0.178	0.094	−0.045
Stress evaluation		–	0.118	−0.239	−0.017	−0.227^*^	−0.132	−0.141	0.016
Professional background									
Professional experience as a homeroom teacher			–	0.041	−0.029	0.274^*^	0.368	0.190	0.041
Weekly hours of teaching				–	−0.048	0.096	−0.176	0.140	0.062
No. of children in the class					–	0.194	0.421^*^	0.217	0.156
Length of experience in teaching						–	0.913^***^	0.560^***^	0.347^**^
Demographic background									
Age of the teachers							–	0.643^***^	0.373^*^
Level of income								–	0.322^**^
No. of individuals in the household									–

***p < 0.001, **p < 0.01, *p < 0.05.

Since about half the teachers in the sample were ultra-orthodox (although the schools belong to the state religious school system), we examined whether religiosity is associated with levels of mindfulness or with stress levels. The findings shown in Table 3 indicate that there is no significant difference in the extent of trait mindfulness by religiosity (*p* < 0.05). In contrast, a difference was found in perceived stress by religiosity [*t*_(85)_ = −2.32, *p* < 0.05]. The data show that ultra-orthodox teachers (*M* = 1.63, SD = 0.61) reported higher perceived stress than did national religious teachers (*M* = 1.28, SD = 0.73).

**Table 3:** Differences in the main research variables by religiosity (*N* = 87).

	National religious teachers (*N* = 46)		Ultra-orthodox teachers (*N* = 41)		*t*_(85)_
	M	SD	M	SD	
Trait mindfulness	3.01	0.46	2.92	0.47	0.883
Stress evaluation	1.28	0.73	1.63	0.61	−2.320*

*p < 0.05. M (Mean). SD (Standard Deviation).

### Research findings in the qualitative study

The thematic analysis (Braun and Clarke, 2012) involved an in-depth familiarization with the text, initial coding of the data, identification of themes, review of the emerging themes, definition and labeling of categories, and the creation of an organized thematic framework while retrospectively addressing the research questions.

The second part, which examines the impact of mindfulness practice on Orthodox female teachers, was conducted using a thematic analysis approach. This method allowed for the identification, analysis, and reporting of patterns emerging from the data.

of the interviews, four main themes emerged regarding the subjective experience of teachers who participated in the “Sagol program” as related to the research questions. The two first themes are a dynamic of distancing: between hidden criticism and open ambivalence, and a dynamic of convergence: independent connections between the world of mindfulness and the world of Judaism. These themes show how teachers who identify as religious negotiate with the secular-scientific world of mindfulness. These negotiations are characterized by a movement between distancing and convergence.

In the first theme, with its focus on the dynamic of distancing, we found that teachers engage in “boundary work” (a sociological term describing the instinctive delimiting aimed at protecting one’s sense of identity and limits). A considerable part (42.85%) of the teachers related that they experience a large distance between their religious world and the world of mindfulness. Their descriptions indicated a sense of distance and a cultural gap between the worlds, which they experienced as existing concurrently. They noted that they lack interfaces and points of connection between the conceptions and practices of the Jewish world and those of the mindfulness world.

Most of the teachers (92.85%) declared, when asked, that they feel no culture sensitivity regarding the mindfulness program. Nevertheless, when asked about connections and similarities between the contents of the course and their religious world and about the need to perform adaptations between the two worlds, their answers revealed cultural sensitivities and mechanisms of both hidden and open criticism. We found that the participants engage in constant “boundary work” between the two worlds, which is partly explicit and partly implicit. The participants encountered the contents of the course, perceived as external to Judaism, through filters that allow them to screen and critique the conceptions and practices taught in the program. Participant 1 emphasized a cautious approach when encountering unfamiliar elements: “Anything that feels foreign makes me take a step back to examine it closely. If I am not sure it aligns with my core values, it becomes another layer of protection.” In her words, Participant 1 describes how she surrounds herself with a protective layer and maintains a safe distance from content she perceives as foreign.

These screening and critical mechanisms are apparently activated automatically, partly consciously and partly unconsciously, among the Orthodox teachers. Their distance is manifested in two main ways: the existence of an initial protective wall versus unknown domains, and the feeling of distance and cultural gap between their own religious world and the world of mindfulness.

Concurrent with the dynamic of distancing, in the second theme that relates to the dynamic of convergence we found that the teachers seek ways of drawing the contents studied in the mindfulness program closer to them through links that they (85.71%) formed independently between their religious world and the world of mindfulness regarding relations with God, interpersonal relations, and religious practices. These links constitute an anchor and a focal point of belonging, like looking at what one has when saying a blessing, equating prayers and meditation, and other connections. As Participant 2 explains: “For example, this story in the morning, we pause for reflection, for gratitude. So, for us, there is prayer, and we begin the morning with prayer. It is a type of meditation, actually. Only if done properly, because even prayer can be recited without truly being present.” The words of Participant 2 reflect the similarity between the emphasis in mindfulness training on full presence in the present moment and a deep examination of various experiences, and the quality of presence required during prayer: intention and focus, which allow for inner connection and full awareness.

The descriptions provided by the interviewees depict God, the Torah, and the religious precepts as an inseparable part of the religious identity.

The third theme indicated the need of teachers for adaptations of the mindfulness world to the Jewish identity, in points of interface and convergence. Most of the teachers (71%) would like such connections to exist to begin with. A fundamental point raised by one of the interviewees, emphasizing the need for adaptations, is related to the rationale of the program, because mindfulness is the training of one’s consciousness, which requires practical experiencing and therefore must be adapted to the target population, their ways of life, worldview, and values unlike an abstract theory that has no real direct connection with one’s life. As Participant 1 emphasizes, “The more points of intersection there are, the more things resonate deeply… The process must align explicitly with the audience.” In this context, Participant 3 further elaborates on the challenge of engaging a conservative audience, noting, “This is an audience where it’s difficult to overcome barriers. It is very conservative. You need to know how to approach it correctly and with proper adaptation.”

In the final part of the qualitative study, we conducted a Grounded thematic analysis for identifying patterns, we examined the association between mindfulness and psychological wellbeing and resilience among Orthodox educators by exploring the impact of a mindfulness-based program that nurtures beneficent qualities through varied mindfulness practices. When examining the interviews, two main themes arose: nurturing inner qualities and the effects on one’s professional and personal life. The first theme, which included cultivating qualities of inner nurturing, was identified among most of the participants and related to the mindfulness practice that allowed them to cultivate qualities of calming, acceptance, inner listening, inner freedom, breathing, release from thoughts, and focusing on the present. In addition, it included cultivating qualities that allow one to cope with challenges and stressful situations, such as the ability to delay one’s reaction, halting the automatic response system, remembering the passing nature of things, focusing on the here and now and on that which exists, work with thoughts and feelings, and the ability to see a wider picture of reality. As Participant 4 states, “Thanks to mindfulness, I learned how to calm myself in the right way, listen to myself, and understand what I am going through. I arrived much calmer, more accepting, and more open. I think this is a process I have gone through.” Similarly, Participant 5 emphasized, “It’s about pausing, listening to my body, to my heart… from practice to practice, it becomes a habit. It’s truly a gift.”

The second theme was found among half the participants and it concerns the impact on one’s professional and personal life, as well as relating to testimonies on the beneficent effects of the mindfulness practice on educators’ interactions, both at school and in the private-family sphere. In this theme we identified another final component among about one-third of the participants, which is support for the teachers’ ability to cultivate psychological wellbeing among their students. One participant (Participant 6) shared, “We see changes in the classroom, where they learn to listen to themselves, to their peers.” Similarly, Participant 7 emphasized, “There are specific things I get to use… the idea of giving them time to relax, all the breathing exercises… so before certain things, this can really help the students, and also personally.

## Discussion

Viktor Frankl states that there is a space between stimulus and response, which allows us to choose our response (Frankl, 1970) (attributed to Viktor Frankl, though not found verbatim in his works).

This study had two aims. The first research aim was to explore whether there is a connection between mindfulness and mental resilience and wellbeing among Orthodox Jewish educators, similar to the findings of other studies in this field. The second aim was to explore whether there is a tension in practicing mindfulness, which originates from the Buddhist tradition, among participants who are Orthodox educators, and whether there is a need for cultural adaptations for religious populations to increase the programs’ effectiveness.

The research findings showed that trait mindfulness and mindfulness practice are associated with increased mental wellbeing and an indirect association with mental resilience.

Trait mindfulness was assessed before the program and was found to be significantly negatively correlated with perceived stress: Teachers with high levels of trait mindfulness reported lower levels of stress, as measured by the Mindfulness questionnaire (FMI; Walach et al., 2006) and the Perceived Stress Scale (PSS; Cohen and Williamson, 1988). To directly assess participants’ subjective experience of stress, the PSS was utilized. This finding suggests that higher trait mindfulness may contribute to enhanced psychological wellbeing by reducing perceived stress, a central factor in overall wellbeing. The relationship between mindfulness and stress reduction is well-established in the literature, where lower stress levels are generally associated with improved psychological and emotional wellbeing. For instance, trait mindfulness has been linked to quality of life (Godfrin and van Heeringen, 2010) and to general psychological wellbeing (Shahar et al., 2010; Shapiro et al., 2008). In addition, people who described good mindfulness competence also reported higher psychological wellbeing and a decline in strain, anxiety, and depression (Hofmann et al., 2010; Carmody and Baer, 2008). Consequently, the reduction of perceived stress through mindfulness practice can be considered a crucial factor in promoting mental health and resilience.

The study also showed that ultra-orthodox teachers reported higher perceived stress than did national religious teachers. In the second part of the study, we examined whether there is cultural sensitivity regarding mindfulness contents. We assumed the existence of cultural sensitivity, as observed in previous studies (Kolsky, 2022; Thomas et al., 2016; Trammel, 2018), but we were unable to find studies that explored this specifically among Orthodox Jewish teachers. Based on previous studies with religious populations (Kolsky, 2022; Thomas et al., 2016), we assumed that in the religious educational field there is a need for cultural adaptations between the worlds. We examined whether the teachers created links independently or whether there is need to create adaptations for them between the content world of mindfulness and Jewish sources.

This study indicates a need for cultural adaptations for the population with which the participants are affiliated. Educators are interested in combining the mindfulness approach and practice in their life routine but need mediation and concrete connections between this world and their Jewish-religious content world.

The findings indicate the necessity of cultural adaptations in the practice of mindfulness among religious educators, as some participants experienced a perceived gap between the principles of mindfulness and their religious worldview. One potential approach to bridging this gap is the integration of relevant Jewish sources within mindfulness practices. This integration could involve the study of texts from Jewish thought, which emphasize introspection, self-awareness, and contemplative practices, thereby aligning mindfulness with familiar religious and philosophical frameworks.

Furthermore, the study’s findings suggest that the identity of the instructor plays a crucial role in the successful implementation of mindfulness among religious populations. Accordingly, training facilitators from within the religious community may enhance both engagement and the overall effectiveness of the intervention. Instructors who share the participants’ cultural and religious background can not only mediate the practice using familiar linguistic and conceptual frameworks but also emphasize aspects of mindfulness that align with Jewish thought and values. Additionally, religiously affiliated instructors may possess a deeper sensitivity to the unique challenges faced by educators in religious settings, thereby fostering a more contextually relevant and culturally responsive mindfulness practice.

The innovative aspect of this study lies in its expansion into a previously unexplored religious stream. The impact of the practice on the teachers is evident (92.85%). They noted the effect of implementing mindfulness on their wellbeing in the professional and personal sphere. This reinforces the connection found in previous studies between implementing mindfulness and psychological wellbeing (Goleman and Davidson, 2017; Hofmann et al., 2010; Kabat-Zinn, 1990; Mandal et al., 2012; Speca et al., 2000). This connection exists among religious teachers, consistent with the findings in previous studies conducted among secular teachers (Emerson et al., 2017; Jennings and Greenberg, 2009; Schussler, 2020).

Based on the study, it may be assumed that cultural adaptations in the content of a mindfulness course would lead to moderation and reduction of the cultural sensitivity that emerged in this study mostly unconsciously. Adapting the intervention can take place on two levels: superficial adaptations (such as adaptations regarding terminology) and more profound adaptations related to the content of the intervention, as informed by the work of Van Mourik et al. (2017).

## Conclusions, limitations, and directions for future research

In conclusion, in an era marked by rapid technological advancement, promoting a life grounded in wellbeing and psychological resilience has become increasingly vital. Orthodox teachers find themselves overburdened, moving between the home on one hand and their work in teaching on the other. The ability to find time for themselves, some degree of space, and being mindful of their body, is critical for them and for their task of educating. In this study, it was found that connecting to Jewish anchors would allow teachers to implement mindfulness practice that facilitates processing and investigating information gathered beyond the boundaries of the mind.

Through a continuous and prolonged process of practice, individuals become less reactive and more consciously responsive to occurrences. Socioemotional skills are based on an educational approach whereby competencies such as listening to others, demonstrating empathy, regulating feelings, and attention can and should be improved. This contention is gradually receiving the support of testimonies from empirical studies in the fields of education, psychology, and neuroscience (Brown and Ryan, 2003; Durlak et al., 2011; Oliveira et al., 2021; Roeser et al., 2013; Tang et al., 2015). Although the study examined the effects of mindfulness over an extended intervention period, it did not include long-term follow-up assessments after the program’s conclusion. Consequently, it remains unclear whether the observed improvements in mental wellbeing are sustained over time. Future research is encouraged to incorporate longitudinal follow-up measures to assess the persistence and durability of mindfulness-related outcomes.

Future research could examine whether there is a difference in the beneficial effects between a generic mindfulness program and one that includes adaptations, incorporating connections to Jewish values, identity, customs, and traditions that align with mindfulness practices.

In the current study, we focused solely on one aspect: mindfulness. Future research could expand to explore the second aspect: compassion (Neff, 2003), and directly examine mental resilience and its significance within a social and cultural context.

## Data Availability

The original contributions presented in the study are included in the article/supplementary material, further inquiries can be directed to the corresponding author.

## Footnotes

^1^The “Purple School” Program is a 3-year initiative, The program is implemented gradually, beginning with the training of a school leadership team and professional development workshops for staff. It then extends to embedding these concepts into the curriculum, daily routines, and structural frameworks of the school, culminating in the establishment of development labs for content adaptation to meet the specific needs and aspirations of the school community. Developed by the Sagol Center for Brain and Mind at the Baruch Ivcher School of Psychology, Reichman University, Herzliya.
